# Cardiac Tamponade; A rare Presentation of Childhood Hypothyroidism

**Published:** 2014-10-30

**Authors:** Sudhir Mehta

**Affiliations:** Department of Pediatrics, Sri Aurobindo Institute of Medical Sciences and PG Institute, Indore, India

**Keywords:** Cardiac Tamponade, Hypothyroidism, Childhood

Myxedema heart disease is a well known entity. Pericardial effusions have been reported in 50-73% of pediatric patients with hypothyroidism in various series^[^^1^^,^^2^^]^, but none of these describes the symptomatic pericardial effusions or cardiac tamponade.

 5 year female child presented with complaints of periorbital puffiness and mild abdominal distension intermittently for last 6 months. She was symptomatically treated with diuretics off and on without much benefit. This time she reported to pediatric emergency with progressively increasing respiratory distress for last 5 days. There was no history of any fever, decreased urine output, orthopnea, or Koch’s contact. She was third live issue of non consanguineous marriage born by normal vaginal delivery following uncomplicated pregnancy. She was immunized for age with no significant family history. She had history of global development delay, development age corresponded to 3 years. On examination she was afebrile with HR of 70/min, RR of 44/min and BP of 80/60 mmHg with pulsus paradoxus. She had coarse facies with dilated neck veins. Her skin was dry and coarse. She had short stature with height less than 3^rd^ centile and normal weight. Her cardiovascular examination revealed silent precordium. Her cardiac border was extending 1.5 cm beyond right sternal margin and 2 cm beyond apex beat on left side on percussion. Her heart sounds were muffled with no murmur. Her abdominal examination revealed free fluid in abdomen. Rest of systemic examination was normal. Chest X ray revealed massive cardiomegaly with CT ratio of 67.8%. Immediate ECHO done on urgent basis, revealed large pericardial effusion with collapse of right atrium during diastole with ejection fraction of 65% and intrinsically normal heart. ECHO guided pericardiocentesis drained 150 ml of fluid. The fluid was clear with 3.3 mg/dl of proteins, 110 mg/dl cholesterol, 20 RBC/µL, 14 lymphocytes/µL, 7 neutrophils/µL and rest of pericardial examination were normal. Rest of hematological and urinary examination was normal. Searching for tuberculosis was also negative. In view of features suggested of hypothyroidism on clinical examination and non-infectious nature of pericardial fluid, her thyroid profile was done which was suggested of hypothyroidism with T3=0.92 nmol/L (1.39-3.7 nmol/L), T4=3.2µg/dl (5.5-12.8µg/dl), TSH=40 mIU/L (0.7-6.4mIU/L). Her anti TPO antibodies were negative and USG neck revealed mild enlargement of thyroid gland. She was started on L-thyroxine with dosage of 4 µg/kg/d. She responded to treatment with thyroid replacement and her pericardial effusion disappeared after 2 months on repeat ECHO. She is on regular follow up and showing improvement in each regards.

 Although mild to moderate pericardial effusions of no clinical significance are frequently reported in pediatric myxedema heart disease, but cardiac tamponade is rare. Literature reports only 2 adolescent girls with cardiac tamponade in association with hypothyroidism, but none has been reported in young children. Author reports a rare case of cardiac tamponade in a young child with previously unrecognized hypothyroidism. Besides bradycardia, minimal pericardial effusions are most common cardiac manifestations of clinical hypothyroidism in adults. Most of pediatric age group patients presenting with this belonged to neonatal period or to Down’s syndrome^[^^1^^,^^2^^]^. Myxedema heart disease was first described by Zondek in 1918 and was completely defined by Fahr in 1925^[^^3^^]^. The pathophysiological derangements responsible for pericardial effusions are increased egress of proteins from blood, decreased lymphatic clearance of proteins and abnormal electrolyte metabolism^[^^4^^]^. At molecular level, commonly observed signs or symptoms in hypothyroidism are attributed to changes in expression of various gene products, which includes alpha myosin heavy chain, beta-1 adrenergic receptors, voltage gated potassium channels and sarcoplasmic reticulum calcium ATPase^[^^5^^]^. The rarity of cardiac tamponade in hypothyroidism is attributed to slow accumulation of fluid and marked distensibility of pericardium^[^^6^^]^. Alexander first described ‘Gold paint effusion’ to describe the golden brown appearance of pericardial fluid due shimmering satin cholesterol crystals^[^^7^^]^. High cholesterol content of pericardial fluid have been attributed to disturbance of lipid metabolism; probably churning action of heart causing precipitation of cholesterol from pericardial fluid. Treatment of cardiac tamponade due to hypothyroidism involves urgent pericardiocentesis followed by thyroxine replacement. Treatment of pericardial effusion with thyroxine replacement leads to resolution of effusion in 2-12 months^[^^5^^]^. In conclusion myxedema heart disease should be suspected in any child with symptoms suggestive of hypothyroidism, presenting with even hemodynamic-ally significant pericardial effusion or cardiac tamponade. Patients with hypothyroidism-associated pericardial effusion should be monitored for development of cardiac tamponade. 
